# Spontaneous resolution of intractable prurigo nodularis after subinsular cortex stroke

**DOI:** 10.1016/j.jdcr.2022.12.008

**Published:** 2022-12-26

**Authors:** Sakeen W. Kashem, Julian C. Motzkin, Kieron S. Leslie

**Affiliations:** a Department of Dermatology, University of California San Francisco, San Francisco, California; b Division of Dermatology, San Francisco General Hospital, San Francisco, California; c Department of Anatomy, University of California San Francisco, San Francisco, California; d Division of Dermatology, San Francisco VA Medical Center, San Francisco, California; e Departments of Neurology and Anesthesia, University of California San Francisco, San Francisco, California

**Keywords:** itch, prurigo nodularis, pruritus, stroke, CNS, central nervous system, IL, interleukin, MRI, magnetic resonance imaging, PN, prurigo nodularis

## Introduction

Prurigo nodularis (PN) is a chronic skin condition characterized by the formation of cutaneous nodules caused by repetitive scratching. Pathogenesis of PN implicates dysregulation of neuroimmunologic circuits that potentiate the itch-scratch cycle.^1^ Development of unilateral PN after contralateral strokes in various brain regions have been reported,^2^, ^3^ suggesting a role of the central nervous system (CNS) in the pathophysiology of PN. Here, we present a case of a patient with a decades-long history of treatment-refractory bilateral PN with a dramatic, spontaneous, and persistent resolution of pruritus and skin nodules after a unilateral subinsular cortex stroke. Findings from this case may further aid in understanding the role of the CNS in the pathogenesis of itch and pruritic skin diseases and could guide potential therapeutic interventions for PN.

## Case presentation

**Fig 1:**
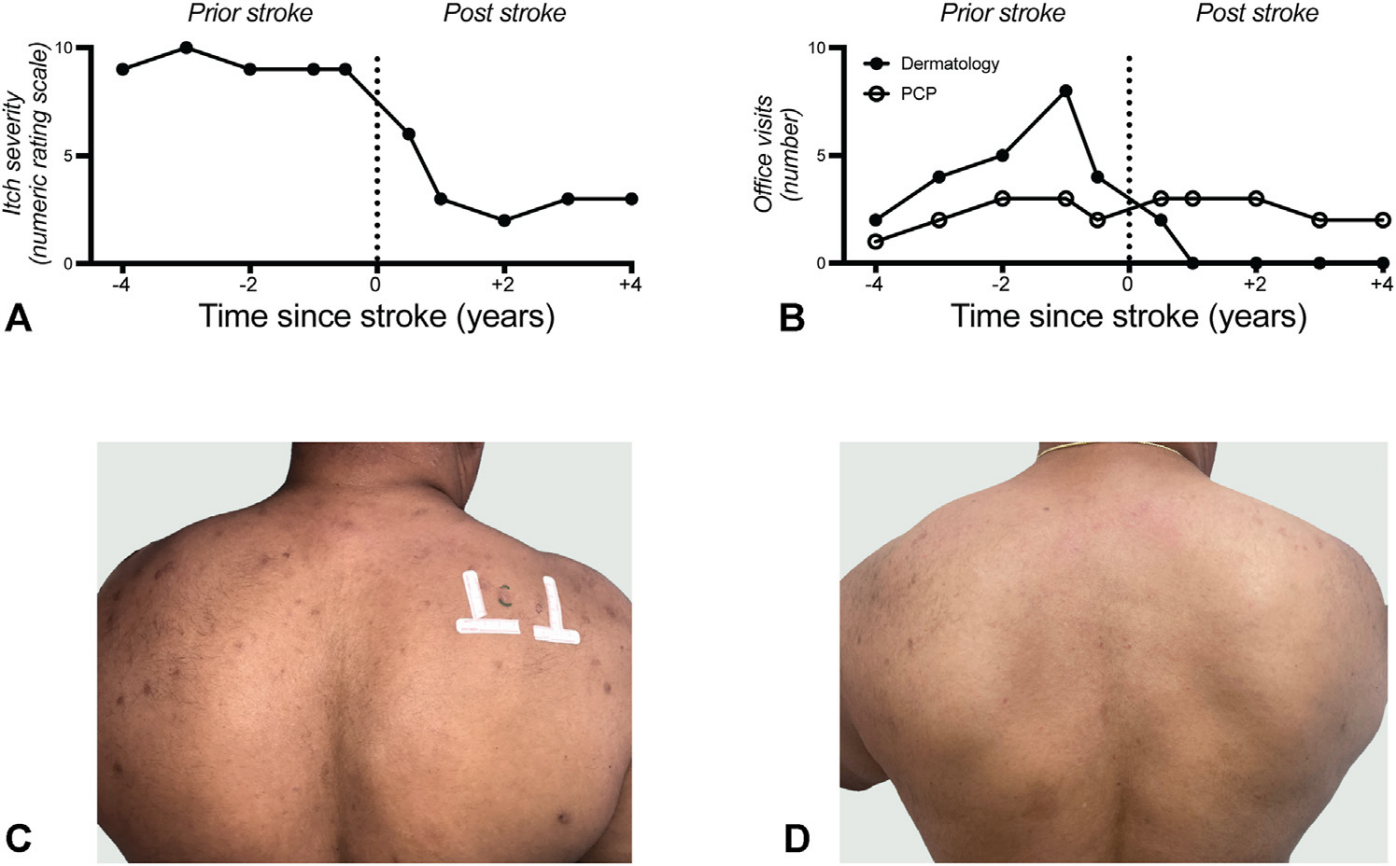
Pruritus severity and dermatology ambulatory visits before and after stroke. **A,** Chronological itch severity represented from 0 (no itch) to 10 (the most severe itch) by patient-reported maximal numeric rating scale in the documented chart and retrospective patient reporting. **B,** Number of office visits per calendar year before and after stroke to dermatology or primary care provider ambulatory services. **C,** Photograph of the patient demonstrating numerous excoriated, lichenified papulonodules on the back sparing the “butterfly” area consistent with prurigo nodularis. **D,** Resolution of lesions and excoriations post stroke in the same patient.

A 56-year-old man presented to the dermatology clinic with a >15 year history of severe pruritus on the bilateral upper portion of the back and neck with >50 excoriated, hyperpigmented, hyperkeratotic, and lichenified papules and nodules consistent with PN. His pruritus was constant at a numerical rating scale of 9 or 10 of 10 and was usually the worst at night with severe sleep disturbances. His medical history was only remarkable for hypertension and prediabetes. An extensive laboratory work-up did not reveal any metabolic, hematologic, toxicologic, autoimmune, or infectious causes. Biopsy results were consistent with PN. Over the following 4 years of regular follow-up in our dermatology clinic, he was seen 21 times and failed treatment with numerous topical medications, as well as phototherapy, neuroleptics, immunosuppression, and antibiotics (Table I, Fig 1, *A* to *D*). Previous authorization for dupilumab and dronabinol treatment was denied and he was considered for initiation of thalidomide therapy for the intractable itch.

**Table I:** Documented pruritus-directed interventions before and after stroke. The list includes all interventions used to treat the patient’s prurigo nodularis 4 years before and 4 years after the stroke

Treatment route	Prior stroke	Post stroke
Topical	Capsaicin	None
	LCD 5% in coal tar	
	Menthol	
	Tacrolimus	
	Triamcinolone, Fluocinonide, Clobetasol	
Intralesional	Triamcinolone	
Phototherapy	Narrowband UV-B	
Systemic	Cetirizine, Fexofenadine	
	Doxepin	
	Doxycycline	
	Gabapentin	
	Ivermectin	
	Methotrexate	
	Mirtazapine	
	Montelukast	
	Naltrexone	
	Prednisone	

*LCD*, Liquor carbonis distillate.

**Fig 2:**
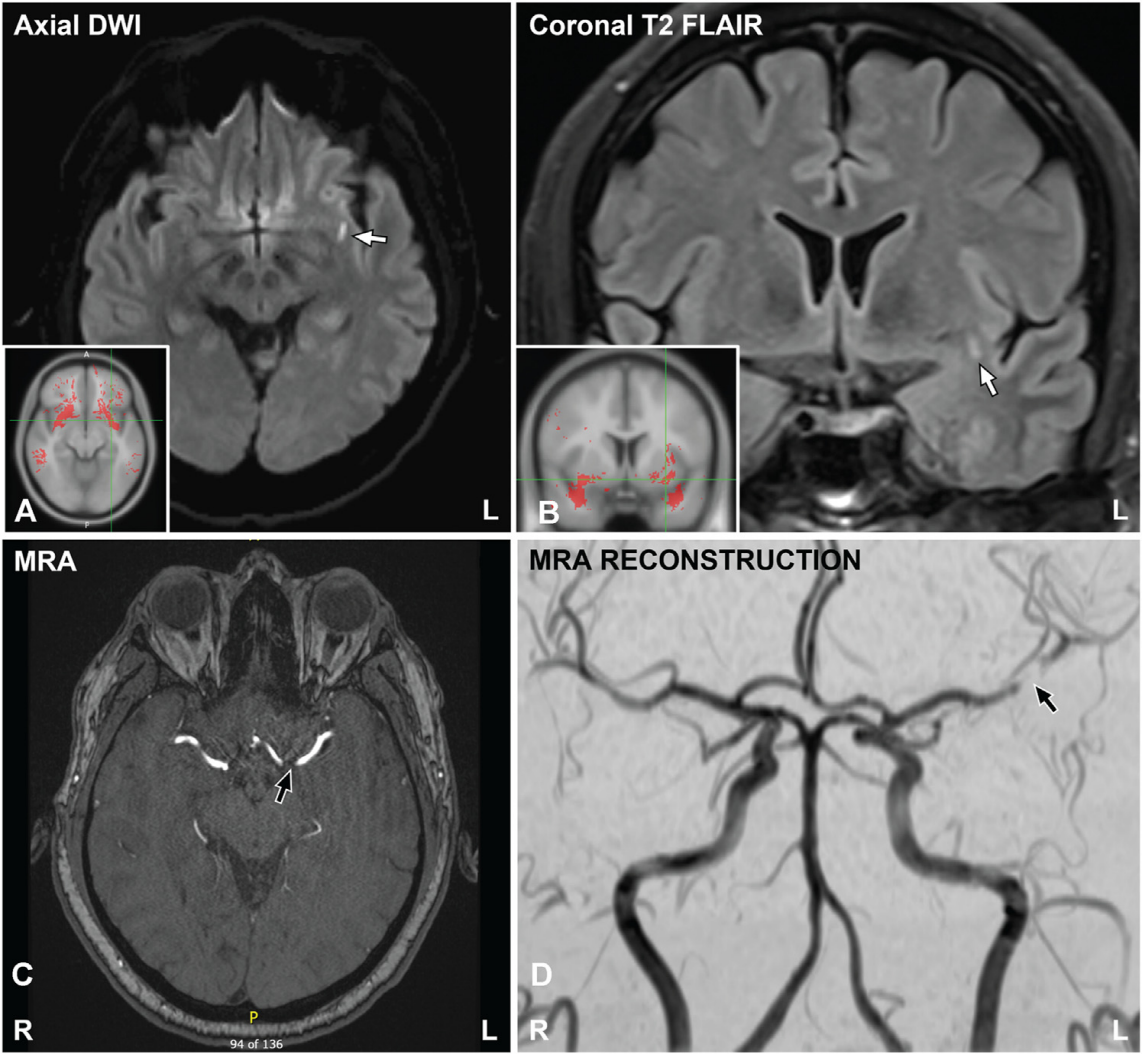
Magnetic resonance imaging and MRA images reveal a subacute left subinsular white matter stroke. **A,** Axial diffusion-weighted imaging shows T2 bright signal in frontal subinsular white matter, consistent with a recent stroke. **B,** The presence of a corresponding bright signal on coronal T2 fluid-attenuated inversion recovery image indicates subacute (>6 days) onset. *Inset*: Johns Hopkins University white matter fiber tract atlas show stroke location on a representative white matter atlas, corresponding with the uncinate fasciculus. **C,** MRA image shows severe stenosis of the middle cerebral artery M2 segment at the origin of the lenticulostriate arteries. **D,** Vessel reconstruction shows filling distal to the area of blockage, suggesting good collateral flow around an occlusion. *FLAIR*, Fluid attenuated inversion recovery; *MRA*, magnetic resonance angiography.

Before thalidomide could be initiated, the patient presented to our emergency department with severe right temporal headaches radiating to the back of his head. The headache would last for 3 to 4 hours and was associated with dysarthria and gradually evolving monocular blurred vision of the right and then left eye. Neurologic examination on admission was normal with no localizing deficits. Magnetic resonance imaging scans demonstrated a small subacute stroke in the left subinsular white matter and magnetic resonance angiography revealed an area of focal stenosis at the proximal left M2 branch of the middle cerebral artery near the origin of the lenticulostriate arteries (Fig 2). His ongoing medications for PN were temporarily held on admission and he was discharged with outpatient follow-up in the Neurology clinic, where he continued to have a normal examination in follow-up.

Two weeks after his stroke admission, he was evaluated in a dermatology clinic and reported near-immediate relief of pruritus and nodules following the stroke. He self-discontinued all PN-directed interventions and the examination revealed healed excoriated nodules on his back. In the 4 years after his stroke, he reported his pruritus severity to be at 0 to 3 of 10, rash to be minimal, and was no longer taking medications for itch or skin inflammation (Table I). No notable nodules or excoriations were noted in the recent photograph. He has not followed up with dermatology for itch but continues to follow up with his primary care provider without notable changes to his health (Fig 1, *B* to *D*).

## Discussion

Here we present a unique case of bilateral PN with complete resolution of pruritus following a small unilateral subinsular stroke. To our knowledge, this is the first such case of improvement of pruritus following a stroke. Below we review current knowledge regarding the pathophysiology and neuroanatomical substrates of PN.

The current understanding of PN implicates both the immune and nervous systems in the pathogenesis of the disease. T cell-derived interleukin 31 (IL-31) is increased in the lesional skin of patients with PN and has been shown to activate subset(s) of dorsal root ganglia neurons expressing the cytokine receptor in both mice and human beings.^4^ Monoclonal antibody against the IL-31 receptor, nemolizumab, has been shown to be effective in treating PN in a randomized clinical trial.^5^ In addition, IL-31 may contribute to aberrant recruitment and proliferation of nerve fibers in the skin of patients with PN, which has been shown to exhibit dermal nerve hyperplasia and decreased epidermal nerve fiber density.^1^ Pruritogenic signals from the skin such as IL-31, IL-4, or IL-13 activate primary afferent fibers that project to the spinal cord dorsal horn, where signals are relayed to the brain to coordinate relevant itch-directed behaviors. Despite the frequent use of CNS neuromodulators to treat PN, whether dysregulation of neuronal signals in the spinal cord or brain can drive itch is unclear.

New onset of central neurogenic itch has been reported following a stroke in multiple brain regions including the brainstem, thalamus, parietal, and frontal lobes.^2^, ^3^, ^6^, ^7^, ^8^, ^9^ Despite variable stroke locations, all prior reports have noted unilateral paralysis and itch after contralateral stroke, leading prior authors to suggest that loss of descending corticospinal or extrapyramidal pathways may contribute to dysregulation of itch percepts.^8^ In 1 case, new onset unilateral PN developed after a contralateral left lateral medullary stroke.^3^ Though symptoms were refractory to neuroleptics, there was significant improvement with IL-4– and IL-13–directed therapy with dupilumab, suggesting that neuroimmune interactions may contribute to PN after central injury.

Our unique case of resolution of refractory bilateral PN after a small unilateral stroke in the frontotemporal white matter is more reminiscent of the “asymbolia” of pain. In previously described cases, patients with pain asymbolia lacked motor and emotional responses to a noxious stimulus despite the acknowledgment of the presence of a pain stimulus. All patients had unilateral insular cortex lesions.^10^ Similarly, our patient acknowledges a persistent low itch severity of 0 to 3 of 10 after his stroke but does not feel enough unpleasantness to scratch or seek treatment. This case may present an instance of itch asymbolia where there is dissociation from the peripheral itch stimuli and the central acknowledgment of unpleasantness that drives scratching. Alternatively, unilateral insular lesions have been shown to lead to spontaneous remission of reward-seeking behavior, and thus, may abrogate the cycle of scratching-induced reward and repetition in the setting of itch. Further studies are needed to precisely pinpoint the specific neuroanatomical substrates and define the neuroimmune interactions that contribute to PN to facilitate the development of new CNS-targeted antipruritic interventions.

## Conflicts of interest

None disclosed.
